# Global Veterinary Diagnostic Laboratory Equipment Management and Sustainability and Implications for Pandemic Preparedness Priorities^1^

**DOI:** 10.3201/eid2904.220778

**Published:** 2023-04

**Authors:** Jennifer N. Lasley, Emmanuel O. Appiah, Kazunobu Kojima, Stuart D. Blacksell

**Affiliations:** World Organisation for Animal Health, Paris, France (J.N. Lasley, E.O. Appiah);; World Health Organization, Geneva, Switzerland (K. Kojima);; Mahidol–Oxford Tropical Medicine Research Unit, Bangkok, Thailand (S.D. Blacksell);; University of Oxford, Oxford, UK (S.D. Blacksell)

**Keywords:** surge capacity, One Health, veterinary, laboratory, equipment maintenance and calibration, quality control, investment, budget and policy, bioterrorism and preparedness, biosafety and biosecurity, management, sustainability, pandemic preparedness, implications for pandemic preparedness, priorities, global veterinary diagnostic laboratory equipment management and sustainability, zoonoses

## Abstract

Substantial investments into laboratories, notably sophisticated equipment, have been made over time to detect emerging diseases close to their source. Diagnostic capacity has expanded as a result, but challenges have emerged. The Equipment Management and Sustainability Survey was sent to the Veterinary Services of 182 countries in mid-2019. We measured the status of forty types of laboratory equipment used in veterinary diagnostic laboratories. Of the 68,455 items reported from 227 laboratories in 136 countries, 22% (14,894/68,455) were improperly maintained, and 46% (29,957/65,490) were improperly calibrated. Notable differences were observed across World Bank income levels and regions, raising concerns about equipment reliability and the results they produce. Our results will advise partners and donors on how best to support low-resource veterinary laboratories to improve sustainability and fulfill their mandate toward pandemic prevention and preparedness, as well as encourage equipment manufacturers to spur innovation and develop more sustainable products that meet end-users’ needs.

The COVID-19 pandemic has highlighted the need for laboratory diagnostics in identifying and characterizing new and emerging pathogens to avoid further spread (*1*). In addition to their role in disease surveillance, laboratories store hazardous pathogens, creating biosafety and biosecurity risks (*2*). Naturally occurring disease outbreaks, laboratory accidents (*3**–**6*), and deliberate releases of pathogens (*7*,*8*) can have severe health (*9*) and economic impacts (*10*) and can greatly disrupt progress toward United Nations Sustainable Development Goals (*11*).

Before the COVID-19 pandemic, there was evidence that many laboratories across sectors faced challenges to their operations and ultimately their sustainability (*12*), such as lack of access to equipment service providers, continuing education for staff, unreliable utilities, and overengineered and poorly adapted infrastructure, to name a few; those shortcomings represent a major challenge for health services worldwide (*13*). The World Organisation for Animal Health (WOAH) Ad Hoc Group on Sustainable Laboratories defines a sustainable laboratory network as one that can continuously deliver specialized services in a manner which is efficient, timely, accurate, consistent, secure, and safe; is in line with international standards and best practices; is provided at an acceptable cost; responds to clients’ needs across sectors (public or private); and benefits One Health goals and the overall One Health system (*14*).

The inability to maintain or improve laboratory performance to leverage investments made by national governments and donors undermines the safety, quality, and security of laboratory activities (*15*). Through its laboratory capacity building and advocacy efforts, WOAH aims to understand and address its member countries’ challenges to sustainability to reduce the risk for biologic escape from veterinary diagnostic laboratories.

We present the results of the WOAH Equipment Management and Sustainability Survey (EMSS) to assess the status of laboratory equipment maintenance, calibration, and repair in veterinary diagnostic laboratories globally. The EMSS sought to quantify the proportion of veterinary diagnostic laboratory equipment that was not properly maintained or calibrated, out of service, and obsolete, as well as access to in-house and local maintenance services and donation of laboratory equipment. Results will enable stakeholders to better understand equipment-related challenges and to inform capacity-building practices, especially in the context of the Pandemic Treaty negotiations and Pandemic Fund investments.

## Methods

The EMSS was distributed to National Focal Points for Veterinary Laboratories and Delegates of all 182 WOAH Member Countries on a rolling basis during May‒August 2019. Nearly 500 questions were asked, and data were collected by using online (Surveymonkey.com) and offline (Microsoft Excel, https://www.microsoft.com) forms in English, Russian, French, and Spanish. The EMSS targeted central veterinary laboratories, defined as the most advanced veterinary laboratory in a country, the national reference laboratory of >1 diseases, often in the administrative capital. Participation was enabled for several national reference veterinary laboratories and other interested veterinary diagnostic laboratories in any country, and the laboratory network level was measured.

We used common reference data and strata (Table 1): World Bank country classifications by income level (which measure gross national income into high, upper middle, lower middle, and low income) (*16*) and WOAH Regional Commission Membership (Africa, Americas, Asia Pacific, Europe, and Middle East) (*17*). 

**Table 1:** Definitions of terms used in Equipment Management and Sustainability Survey conducted by World Organisation for Animal Health, 2019

Term	Definition
Donated equipment	Equipment that was given by a partner for an unlimited amount of time and belongs to the beneficiary laboratory
Equipment	Critical laboratory tools and machines for basic and essential veterinary laboratory diagnosis and analysis
External service provider	A service provider outside a country
High-income country	>12,535 Gross National Income per capita in United States Dollars 2020, as defined by World Bank Income Level Index
Improperly/not properly calibrated equipment	Equipment to which precise adjustments have not been made to ensure accurate measurement for a particular function and to establish the metrological traceability of the reported results
Improperly/not properly maintained equipment	Equipment for which preventive maintenance has not been conducted in accordance with a specified time schedule, involving functional checks and servicing, and replacement of consumables
In-house service provision	Equipment maintenance and calibration tasks assigned to and conducted by existing laboratory employees
Local service provider	A service provider within a country
Low-income country	<1,035 Gross National Income per capita in United States Dollars 2020, as defined by World Bank Income Level Index
Lower‒middle-income country	1,035‒4,045 Gross National Income per capita in United States Dollars 2020, as defined by World Bank Income Level Index
Malfunctioning	Equipment that is not working properly, which might require maintenance, repair, or calibration
Out-of-service equipment	Equipment that is not being used because it is not working properly
Repair	Corrective maintenance performed after failure or detection of a fault, to restore equipment to working order, including repairing or replacing parts of the equipment
Upper‒middle-income country	4,045–12,535 Gross National Income per capita in United States Dollars 2020, as defined by World Bank Income Level Index
World Organisation for Animal Health regions	Africa, Asia Pacific, Europe, Middle East, Americas, as defined by the World Organisation for Animal Health

For the survey, maintenance was defined as actions carried out on a specified schedule, involving functional checks and servicing, and replacement of consumables. Calibration was defined as precise adjustments made to laboratory equipment to ensure accurate measurement for a particular function and to establish the metrological traceability of the reported results.

Results are based solely on self-reporting. Although equipment inventories were not directly accessed, a list of 40 common types of veterinary laboratory equipment was used, focusing on critical items for basic and essential veterinary laboratory analysis. Infrastructure and premises status were not included in the survey. We provide the original survey (Appendix); full dashboards and detailed figures for all data reported are available on the WOAH website (https://www.woah.org/en/what-we-offer/emergency-and-resilience/sustainable-laboratories/laboratory-equipment-management-and-sustainability).

## Results

### Responses

Of 182 WOAH member countries, 136 (75%) countries were represented, and detailed data were received from 223 veterinary laboratories from all 5 WOAH regions. The largest proportions of represented countries (30%, 41/136) and participating laboratories (45%, 101/223) were in Europe. Most responses were from central veterinary laboratories (58%, 129/223). The proportion of nonresponse was relatively consistent across regions (range 23%–36%) and World Bank income levels (range 24%–42%).

### Overall Equipment Reported

Laboratories reported 68,455 items. Of those items, monochannel and multichannel pipettes (44.5%, 30,528/68,455) were the most frequent, followed by incubators (7.5%, 5,155/68,455), refrigerators (7%, 4,823/68,455), agitators (6.8%, 4,671/68,455), freezers (5.8%, 3,994/68,455), centrifuges (4.5%, 3,076/68,455), and biosafety cabinets (4.3%, 2,965/68,455). Most (74.6%, 51,047/68,455) items were in high-income countries. Europe reported 65.4% (44,747/68,455) of total equipment reported. Most equipment reported (76.5%, 52,355/68,455) was at the central level. Equipment inventory reviews were performed at least annually by 88.8% (190/214) of laboratories. Given that some equipment does not require calibration, those items were removed from the denominator for all calibration measurements.

### Equipment Maintenance and Calibration

**Figure 1:**
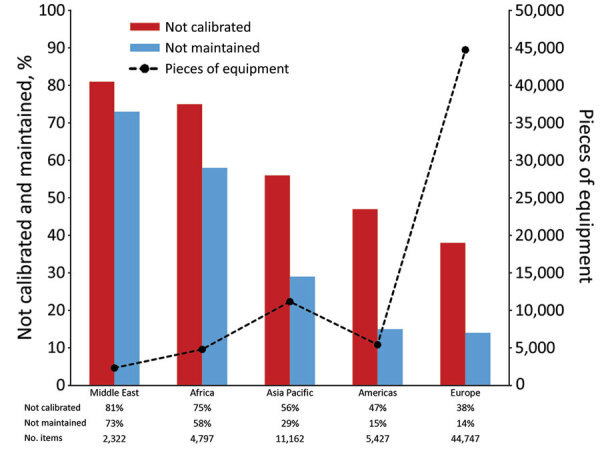
Reported laboratory equipment and proportion not properly calibrated or maintained, by World Organisation for Animal Health region, for Equipment Management and Sustainability Survey conducted by World Organisation for Animal Health, 2019.

**Figure 2:**
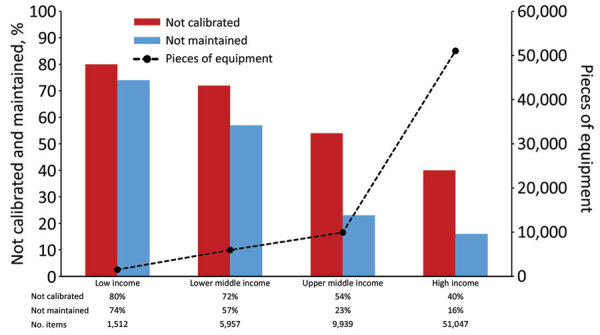
Reported laboratory equipment and proportion not properly calibrated or maintained, by World Bank income level, for Equipment Management and Sustainability Survey conducted by World Organisation for Animal Health, 2019.

Overall, 21.8% (14,894/68,455) of equipment was not properly maintained, and 45.7% (29,957/65,490) was not properly calibrated. Regional differences were observed in improperly maintained equipment (Figure 1). High-income countries reported the largest amount of equipment and the lowest proportion of improperly maintained (15.8%, 8,044/51,047) and calibrated (38.4%, 19,607/51,047) equipment (Figure 2). However, an inverse trend was observed in the lowest-income countries. Laboratories in low-income countries had the smallest amount of equipment (1,512) but had the highest proportions reported as improperly maintained (74.1%, 1,120/1,512) and calibrated (80.5%, 1,157/1,438).

Noncompliance with maintenance protocols based on self-report for different types of equipment ranged from 14.1% (pipettes, 4,296/30,528) to 42.9% (roller systems, 27/63) (Table 2). Given that pipettes represented 44.6% (30,528/68,455) of equipment reported and could bias overall results, when they were excluded from the analysis, the proportion of equipment that was improperly maintained (21.8% [14,894/68,455]) increased to 27.9% (10,598/37,927). Similarly, the proportion of equipment that was improperly calibrated (45.7% [29,957/65,490]) increased to 48.1% (16,825/34,962).

**Table 2:** Reported equipment not properly maintained by equipment category for Equipment Management and Sustainability Survey conducted by World Organisation for Animal Health, 2019*

Laboratory equipment	Africa	Americas	Asia Pacific	Europe	Middle East	Global
Roller system	85%	50%	20%	34%	0%	43%
Distillator	66%	23%	36%	29%	66%	41%
BSC class II A1	74%	31%	40%	26%	83%	40%
Magnetic agitator	66%	9%	29%	47%	61%	39%
Water bath	62%	23%	29%	36%	87%	39%
Microtome	71%	19%	36%	23%	89%	38%
Trichinoscope	67%	100%	21%	37%	50%	35%
Vortex	58%	11%	32%	33%	88%	35%
Autoclave	63%	16%	36%	24%	61%	34%
Gas incubator	54%	74%	25%	14%	54%	34%
Microplate reader	60%	14%	37%	26%	70%	34%
Dark-field microscope	73%	8%	38%	24%	50%	33%
Mixer jar	69%	38%	22%	28%	67%	31%
Oven	53%	17%	37%	14%	83%	31%
Colony counter	52%	29%	34%	19%	100%	30%
Ovoscope	100%	38%	37%	13%	100%	30%
Freezer	66%	4%	23%	25%	75%	29%
pH meter	83%	20%	26%	16%	58%	29%
Gel documentation	52%	21%	29%	24%	57%	28%
Refrigerator	59%	13%	31%	21%	64%	28%
Shaker	65%	14%	21%	26%	57%	28%
Spectrophotometer	51%	24%	19%	26%	58%	28%
Vacuum pump	70%	13%	32%	20%	58%	28%
Fluorescent microscope	47%	21%	26%	19%	67%	26%
Fume hood	44%	29%	43%	16%	92%	25%
Microplate washer	70%	13%	22%	17%	72%	25%
Water filtration	71%	24%	33%	11%	55%	25%
Centrifuge	54%	14%	35%	12%	66%	24%
Plate shaker	52%	11%	31%	12%	78%	24%
Real-time PCR	33%	19%	29%	16%	75%	24%
Inverted light microscope	61%	9%	33%	16%	71%	23%
Microscope	53%	17%	22%	13%	76%	23%
Incubator	45%	15%	31%	16%	61%	22%
Conductometer	67%	13%	25%	10%	56%	21%
Transilluminator	65%	9%	29%	13%	56%	21%
Thermal cycler	64%	12%	20%	12%	55%	19%
Biological safety cabinet class II A2	41%	40%	25%	8%	88%	18%
Biological safety cabinet class I	57%	31%	57%	6%	67%	17%
Electrophoresis	49%	6%	28%	8%	36%	15%
Pipette	57%	8%	26%	8%	77%	14%

Calibration was reported as a larger problem than improper maintenance. Although the lowest levels of improper calibration were in Europe, there was a range of noncompliance with calibration protocols within that region, from 25.2% (real-time PCR, 152/603) to 54% (centrifuges, 1,660/3,076) (Table 3) across different types of equipment.

**Table 3:** Reported equipment not properly calibrated by equipment category for Equipment Management and Sustainability Survey conducted by World Organisation for Animal Health, 2019

Laboratory equipment	Africa	Americas	Asia Pacific	Europe	Middle East	Global
Centrifuge	73%	66%	79%	37%	79%	54%
Fume hood	76%	59%	63%	39%	92%	49%
Spectrophotometer	70%	59%	81%	29%	75%	48%
Pipette	69%	13%	39%	44%	80%	43%
pH meter	82%	78%	59%	14%	50%	42%
Microplate reader	76%	40%	51%	22%	80%	41%
Autoclave	77%	36%	62%	14%	77%	40%
Conductometer	67%	50%	33%	12%	67%	28%
Thermal cycler	77%	39%	35%	12%	70%	28%
Real-time PCR	52%	44%	33%	12%	64%	25%

### Equipment Service Providers

**Figure 3:**
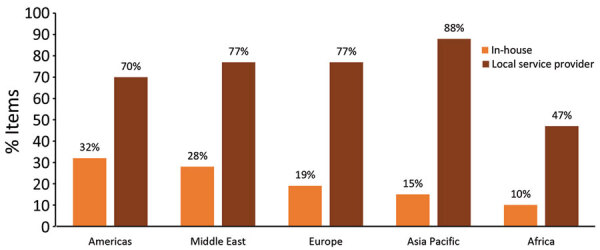
Reported availability of in-house and local service providers for laboratory equipment, by World Organisation for Animal Health region, for Equipment Management and Sustainability Survey conducted by World Organisation for Animal Health, 2019.

**Figure 4:**
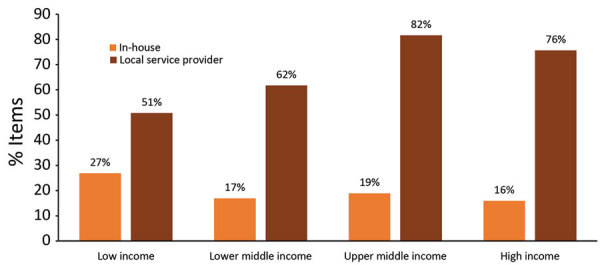
Reported availability of in-house and local service providers for laboratory equipment, by World Bank income level, for Equipment Management and Sustainability Survey conducted by World Organisation for Animal Health, 2019.

Results from the EMSS indicated that the main reported barriers to maintenance were expensive services, insufficient budget allocation, and no local (i.e., within one’s country) service providers available. Globally, competencies to maintain and calibrate existed in-house for 18% of each type of equipment and locally for 74%. In Africa, competencies to maintain and calibrate existed in-house for only 10% of equipment and locally for 47% of equipment (Figures 3, 4). The largest proportion of in-house expertise (27%) and the smallest proportion of local service providers (51%) was in low-income countries and did not translate to higher maintenance and calibration compliance levels. A total of 69% of laboratories were satisfied with local service providers, compared with 58% with external (e.g., outside of a country) service providers.

### Out-of-Service and Malfunctioning Equipment

Globally and for all equipment combined, 10.8% (7,394/68,455) was out of service. Laboratories in low- and lower-middle income countries reported that 25.8% (390/1,512) were out of service, compared with 15.5% (1,536/9,939) of upper-middle income countries and 7.6% (3,905/51,047) of high-income countries. Pipettes represented the largest proportion of out-of-service equipment. However, when they were removed from the dataset, the global out-of-service rate increased to 14.4% (5,479/37,927), suggesting that pipettes are in a slightly better condition than other equipment. Globally, the top 3 reported causes of malfunction were overuse, software, and electricity problems, such as voltage incompatibility (i.e., 120V to 220V), power surges, inconsistent electricity, and subsequent wear on electrical components. However, in Africa, the leading causes of malfunction reported were delayed maintenance, electricity problems, and overuse. The 3 most frequent ways that obsolete, damaged, or outdated equipment was managed were labeled out-of-service, placed in storage, and isolated in the laboratory without labeling.

### Donated Equipment

**Figure 5:**
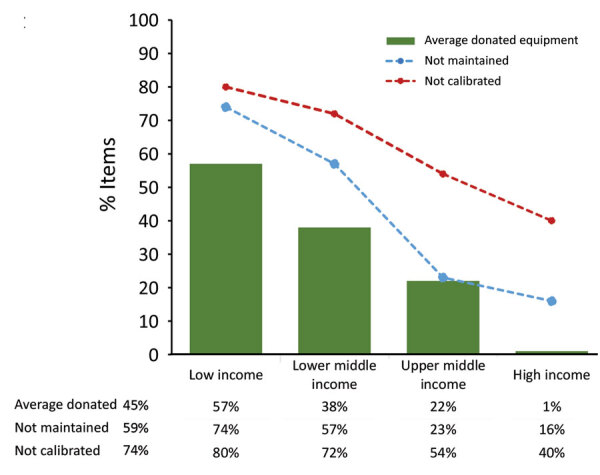
Reported estimated proportion of donated equipment and proportion not properly maintained or calibrated, by World Bank income level, for Equipment Management and Sustainability Survey conducted by World Organisation for Animal Health, 2019.

**Figure 6:**
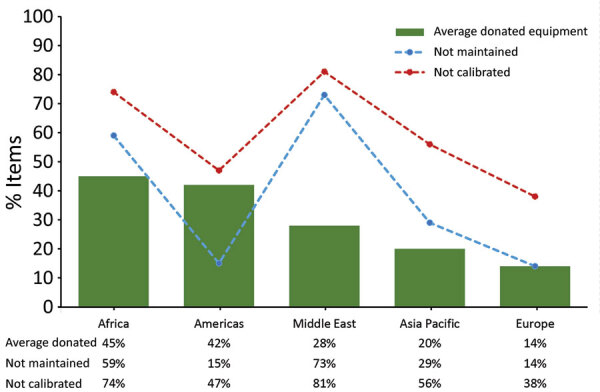
Reported estimated proportion of donated equipment and proportion not properly maintained or calibrated equipment, by World Organisation for Animal Health region, for Equipment Management and Sustainability Survey conducted by World Organisation for Animal Health, 2019.

Globally, 48.8% (105/215) of laboratories reported donated equipment in their laboratory and estimated on average that 30% was donated. Low-income countries estimated on average that 57% were donated, followed by lower-middle (38%), upper-middle (22%), and high (1%) income countries (Figure 5). The percentage of reported estimated donated equipment varied by region, with Africa the highest (45%), followed by the Americas (42%), Middle East (28%), Asia (20%), and Europe (14%) (Figure 6).

## Discussion

The EMSS results describe the status of equipment in veterinary diagnostic laboratories globally and highlight the difficulties relating to the sustainability of these laboratory networks (Table 4). We expect the results represent the tip of the iceberg in the veterinary laboratory sector. Although we are aware of no equivalent study in human health or clinical laboratory settings, equipment-related challenges, such as limited local capacity and capability and sustainable resourcing, are common to both sectors (*12**,**18**–**23*). Estimates suggest that ≈40% of medical equipment in the hospital setting in developing countries remains out of service, predominantly because of a lack of infrastructure, training, and maintenance (*24**–**27*). Given the smaller number of veterinary laboratories compared with human health laboratories, we expect that the equipment management and sustainability challenges in human health laboratories are likely more pronounced.

**Table 4:** Key results from the Equipment Management and Sustainability Survey conducted by WOAH, 2019*

Results
• A total of 136 (75%) of 182 WOAH Member Countries responded; detailed data were received from 223 veterinary laboratories in all 5 WOAH Regions,
• A total of 68,455 items of laboratory equipment were reported with an approximate value of 264.4 million €.
• Globally, 22% of the equipment reported was not properly maintained, and 46% was not correctly calibrated.
• Low-income countries had the smallest proportion of equipment but had the highest proportions of improperly maintained (74%) and calibrated (80%) equipment.
• Globally, competencies to maintain and calibrate equipment existed in-house for 18% of equipment and within one’s country for 74% of equipment. In Africa, competencies to maintain and calibrate equipment existed in-house for only 10% of equipment and within one’s country for 47% of equipment.
• Globally and for all laboratory equipment combined, 11% of equipment reported was out of service.
• Low-income countries estimated that 57% of their equipment was donated, followed by lower-middle (38%), upper-middle (22%) and high (1%) income countries.
• Full analysis available at https://www.woah.org/en/what-we-offer/emergency-and-resilience/sustainable-laboratories/laboratory-equipment-management-and-sustainability/

*WOAH, World Organisation for Animal Health.

The study results provide compelling evidence to formulate strategies toward building and sustaining veterinary laboratory capacity over time. Those strategies include long-term planning; balancing investments from capital to operating expenses; balancing proportions of investments across internal, external, and revenue sources; and setting priorities to maintain strengthened infrastructure and capability, especially in low-resource settings. Given the prevalence of donated equipment, the undesirable proportions of equipment in poor condition, and the uneven access to local service providers in the veterinary laboratory setting, this study has demonstrated that laboratory equipment has become a consumable commodity that can be readily replaced by partners when large (or small) equipment management challenges are encountered, instead of valuable and valued capital investments to be leveraged over time by national authorities and partners.

Therefore, creative solutions are required to deal with equipment, consumables, waste, and compliance, which might involve public‒private partnerships, cost-sharing arrangements, and other innovative approaches (*28*). Proposed actions address equipment management challenges at the laboratory level (Table 5) and key implications for capacity-building policy and practice (Table 6).

**Table 5:** Actions laboratories can take to address sustainability challenges through improved equipment maintenance and calibration for Equipment Management and Sustainability Survey conducted by World Organisation for Animal Health, 2019

Action
Prioritize the equipment used most, with a particular focus on equipment needed most in an emergency to detect emerging diseases, such as African swine fever, African horse sickness, avian influenza, and coronavirus disease
Check annual operating budgets for equipment maintenance and calibration resources
Plan how to mobilize resources
Act to mobilize resources
Make/update list of calibration and maintenance service providers by equipment type, ready for an emergency
Offer calibration or preventive and corrective maintenance services to neighboring laboratories, if capacity exists
Train neighboring laboratories to conduct calibration or preventive and corrective maintenance, if capacity exists
Perform preventative maintenance on prioritized equipment without delay.
Plan the next check of prioritized equipment, and then do it on a regular basis
Train staff on proper preventative maintenance of prioritized equipment
Cultivate relationships with service providers
Have prioritized equipment calibrated without delay
Plan the next calibration verification of prioritized equipment, and then do it on a regular basis
Train staff to calibrate simpler prioritized equipment
Cultivate relationships with service providers
Have prioritized equipment repaired without delay
Plan the next check and calibration of prioritized equipment, and then do it on a regular basis
Train staff to do simple repair of prioritized equipment
Cultivate relationships with service providers
Perform equipment inventory review without delay
Plan the next equipment inventory and act to conduct on a regular schedule

**Table 6:** Key findings and implications on capacity building policy and practices affecting sustainability for Equipment Management and Sustainability Survey conducted by World Organisation for Animal Health, 2019

Findings
• Laboratory equipment has become a consumable commodity that can be readily replaced by partners in case of management challenges, instead of valuable and valued capital investments to be leveraged over time by national authorities.
• Laboratory equipment management is a One Health, cross-sectional, and cross-sectoral issue, often affected by a lack of coordination and overinvestment in capital resources like equipment in the laboratory sector.
• No equivalent study has been performed in public health or clinical laboratory settings, but equipment-related challenges, like limited local capacity and sustainable resourcing, are common to all health laboratories.
• High rates of uncalibrated equipment do not provide confidence in laboratory results, calling into question the intrinsic value of the testing performed and the return on investment.
• Poor equipment maintenance and calibration threaten safety, security, business continuity, quality, accuracy, and timeliness of results, with a measurable impact on human health, animal health, and environmental health, and, therefore, people’s livelihoods and economies.
• The proliferation of high-containment laboratories in locations where specialized equipment and infrastructure services are difficult or impossible to access has meant that donation recipients neither have the financial nor human capital to maintain the laboratory and its equipment, leading to inevitable engineering failures and increasing the potential for inadvertent laboratory releases of dangerous pathogens.
• New strategies are needed to sustain capacity built, including long-term planning, balancing investments from capital to operating expenses, and setting priorities to maintain strengthened infrastructure and capability, especially in low-resource settings, which may involve public-private partnerships, where local business and expertise are supported through subsidies, incentives, or agreements to encourage the development of local service provision and not just on in-house expertise.
• Partners of health laboratories must agree on more rigorous, evidence-based, best practices and standards.
• Demand for maintenance and calibration services across the health laboratory sector is large and should be consolidated to support local service providers by cost sharing and bulk ordering.
• Investment from the national government and private sector will be required in the One Health context.
• The waste of precious resources should be met with innovative and pragmatic solutions that focus on getting back to management basics, rational supply and demand thinking, and building coherent systems that are appropriately sized and fit for purpose.
• Given that challenges encountered in veterinary laboratories are described and agreed to be similar in public health laboratory settings, action aiming toward sustainable health laboratory systems in the One Health space to improve pandemic preparedness is needed.
• Organizations that invest in laboratory capacity building or strengthening may hold similar, although unexploited, data and could provide precise and robust measurements along these same metrics across health sectors.

The lowest income countries have the least amount of equipment, but they have the most difficulty properly maintaining and calibrating increasingly complex equipment. Improving availability and access to local service providers is an opportunity to address this long-lasting and pervasive problem. Higher satisfaction reported in the EMSS with local service providers suggests an opportunity to develop this market without sacrificing quality. This development could be addressed through public‒private partnerships in which local businesses and expertise are supported through subsidies, incentives, or agreements to encourage the development of local service provision and not just in-house expertise, which is affected by staff attrition and brain drain. Demand for those services across the health laboratory sector is large, in animal health, human health, clinical, environmental or food safety laboratories, and should be consolidated to support local service providers. To support that strategy, One Health investment from the national government and private sector will be required.

Developing local capacity, as in the case of biosafety cabinet certification in Southeast Asia (*29*), is the ideal option and is the target of many development efforts, but it has been challenging to sustain locally because it is too often reliant on external donor funding, lacking sustainable domestic financing. Developing in-house and local capacity is adversely affected by brain drain because skills are attractive and in demand across sectors. However, given the risks of emerging disease outbreaks and pandemics, as demonstrated by COVID-19, governments’ investment in this resource is critical to ensuring that laboratory systems are sustainably resourced, prepared, and equipped to face future challenges (*1*).

Accreditation of quality management systems (QMS), with a focus on the International Organization for Standardization/International Electrotechnical Commission (ISO/IEC) 17025:2017 (https://www.iso.org/isoiec-27001-information-security.html) for testing and calibration laboratories, has become an ideal for veterinary laboratories worldwide (*30*). Laboratories in the human health sector have made great strides in achieving QMS accreditation to international standard ISO/IEC 15189:2012. Accreditation of a diagnostic laboratory requires 3 components: independent or third-party assessment; suitably validated tests performed by proficient laboratory operators in an adequately equipped laboratory; and ongoing internal and external quality control. Those components provide confidence in test outcomes and demonstrate competency and ability to produce technically valid diagnostic results that meet the needs of customers and decision-makers involved in health and surveillance programs (*30*). Incumbent on the success of QMS in diagnostic laboratories is the ability to calibrate equipment to meet the requirements of the ISO/IEC standards, thus exacting an economic effect on low-income countries. Our results indicate a lack of local calibration expertise, insufficient resources to cover costs, and an unwillingness to implement QMS. Nevertheless, high rates of uncalibrated equipment, especially in limited resource settings, do not provide confidence in the laboratory results, calling into question the intrinsic value of the testing performed and the return on investment. Therefore, it is incumbent on international organizations, funding agencies, and national regulatory agencies to demand that all laboratories producing diagnostic results meet international QMS requirements.

Equipment donation provides critical support to laboratories in low-resource settings. Unfortunately, the issue of donated equipment is fraught with good intentions. Donated equipment requires sufficient funds to ensure maintenance, calibration, repair, and replacement, but those funds are not usually built into national laboratory budgets. Partners often make decisions in isolation without proper consultation with the end users and national authorities (*31*). There is the potential, therefore, that the item does not meet the end users’ needs or match their environmental realities. Furthermore, partners often purchase equipment without consideration of available local service provision and availability. In the event of failure, repair might not be locally available, making the item unusable when it might have been easily and cheaply repaired elsewhere. It is acknowledged by partners that most of these national laboratories rely on external aid to function and may be unsustainable (*32*).

Paradoxically, resources provided to low-income and middle-income countries in the decades before COVID-19 enabled laboratories in those countries to be better equipped than ever before to join the response to COVID-19. All laboratory-system partners should agree on more rigorous standards for evidence-based best practices for equipment donation. Guidelines have been developed by the World Health Organization in medical equipment donation (*33*); adaptation of these guidelines to the laboratory setting across sectors in a One Health approach is required and should be adopted by all partners. It is therefore necessary to learn from this study, examine gaps in capacity, and ensure that donors and international organizations avoid providing support on an individual or haphazard basis but do so in a coordinated manner that is ongoing, purposeful, and contiguous.

A perverted result of equipment donation to low-resource laboratories is the accumulation of malfunctioning and obsolete equipment in the laboratory itself. This study demonstrated that 96% of reported nonfunctional equipment was labeled out of service on the bench, put into laboratory storage, or isolated in the laboratory. This equipment therefore goes unused, is useless, and contributes to the electronic waste (or e-waste) disaster for human, animal, and environmental health, reaching 2.9 million tons in 2019 in the Africa Region, according to the Global E-waste Monitor 2020 (*34*). Laboratory e-waste has processed dangerous and infectious pathogens, thus adding increased safety and security risks. Efforts to reduce waste of precious resources should be focused on innovative reselling, refurbishing, recycling, and repair schemes across laboratory sectors to reduce waste, creating a do-it-yourself culture of preventive and corrective maintenance by using social media, promoting electronics right-to-repair communities, and engaging manufacturers to support those initiatives.

In the context of the COVID-19 pandemic, veterinary laboratories played a critical role by providing surge capacity for the human health sector for diagnostic testing response (*35**,**36*). As found in the EMSS, in veterinary laboratories, 175 (11.6%) of 1,513 pieces of equipment for conventional and real time PCR were out of service globally as of August 2019. That lost surge capacity is tangible, given the pressure on this technology during the COVID-19 pandemic. A postpandemic follow-up study to determine the effect of lost capacity attributed to poorly maintained equipment would provide valuable insights. The pandemic has also created worldwide waves of shortages of critical supplies, parts, and materials needed to support complex machinery; the laboratory sector will continue to be adversely affected by those critical shortages.

Given the enormity of this global project and the requirements for providing in-depth data over a lengthy questionnaire, there is the potential for inaccuracy, including recall bias, nonresponse, and social acceptability. However, despite those limitations, a high response rate (75%) was recorded, and 79% of laboratories completed the full survey, demonstrating a high degree of stakeholder interest.

A total of 93% of respondents worked in laboratory settings at the time of the survey and therefore were best placed to respond accurately to the questionnaire. The response rate was similar across different groups (i.e., World Bank Income Level, WOAH region), providing relative accuracy across strata. Despite the inherent limitations of a large-scale survey, the information received from the respondents was considered reliable. However, following up on the results of this questionnaire as standard practice for any laboratory capacity building project will be imperative to confirm the results, build on the knowledge base, and monitor progress.

Much can be learned about equipment management and sustainability from the EMSS about veterinary laboratories. Given that challenges encountered in veterinary laboratories are largely described as and agreed to be similar in human health laboratory settings, results should initiate much-needed research, discussions and action aiming toward sustainable laboratory systems in the One Health space to improve pandemic preparedness across sectors. These results represent the first attempt on a global scale to determine the status of equipment in the public veterinary diagnostic laboratory setting. Furthermore, organizations that invest in laboratory capacity building or strengthening may hold similar, although unexploited, data and could provide precise and robust measurements along these same metrics across health sectors.

Veterinary laboratories attract investments from stakeholders, including security, human health, One Health, agriculture, trade, and development. Those stakeholders are interested in the best outcomes for laboratories and their smooth management. Laboratory equipment management is a One Health, cross-sectional, and cross-sectoral issue, often affected by a lack of coordination and overinvestment in capital resources such as equipment in the laboratory sector. Poor equipment maintenance and calibration threaten safety, security, business continuity, quality, accuracy, and timeliness of results, with a measurable effect on human health, animal health, and environmental health, and, therefore, on livelihoods and economies. The value of the global laboratory equipment and consumables market across all sectors is estimated at US $30.6 billion in 2020 (*37*) which forces reflection on how to mitigate the challenges encountered by end users and countries who benefit from external investment at all levels and across all sectors.

Results from the EMSS will contribute to WOAH integrating key performance indicators for laboratory equipment into its capacity-building programs and strict limits on provision of equipment, with the expectation that its partners will follow suit when considering further donation and investment in laboratory capacity building. Additional partners should join the efforts of international organizations such as WOAH and the World Health Organization to sensitize key donors and to build consensus that equipment donation should be tied to an achievable installation, calibration, and maintenance plan with a secured, long-term budget commitment, by either the donor or preferably the recipient government.

The current global context has demonstrated that laboratory preparedness is essential and that regular laboratory equipment maintenance and calibration are critical. Veterinary laboratories played a crucial role in the COVID-19 pandemic, armed with their experience in combatting outbreaks among large populations (such as for highly pathogenic avian influenza), in providing expertise on disease origin and evolution, population medicine, scientific research on the susceptibility of animals, surge capacity for the human health sector, and human antibody response to vaccination. We expect that our findings will inform national authorities to understand the challenges they face related to equipment management and to better plan to sustain investments made by resource partners; influence partners to reconsider investments in light of the sustainability challenges faced by national authorities and to design effective investments based on real needs of laboratories; and encourage manufacturers, researchers, innovators, and engineers in their efforts toward effective and more sustainable designs that are fit-for-purpose in low-resource settings.

Appendix Additional information on global veterinary diagnostic laboratory equipment management and sustainability and implications for pandemic preparedness priorities.
