# Supplementary material for: Global Veterinary Diagnostic Laboratory Equipment Management and Sustainability and Implications for Pandemic Preparedness Priorities
Source: Emerg Infect Dis. 2023 Apr;29(4):e220778. doi: 10.3201/eid2904.220778 (PMC10045690; doi:10.3201/eid2904.220778)
Supplement: Appendix — Additional information on global veterinary diagnostic laboratory equipment management and sustainability and implications for pandemic preparedness priorities. [file 22-0778-Techapp-s1.pdf]

Article DOI: https://doi.org/10.3201/eid2904.220778

EID cannot ensure accessibility for supplementary materials supplied by authors. Readers who have difficulty accessing supplementary content should contact the authors for assistance.

Global Veterinary Diagnostic Laboratory Equipment Management and Sustainability and Implications for Pandemic Preparedness Priorities

Appendix

The following pages show the WOAH Equipment Management and Sustainability Survey that was used in 2019 to assess the status of laboratory equipment maintenance, calibration, and repair in veterinary diagnostic laboratories globally.

Laboratory Equipment Status Questionnaire for Maintenance/Repair/Calibration - Part I

Background

The main objective of the survey is to assess the status of laboratory equipment maintenance, repair, and calibration for the purposes of a policy paper on “Investment Needs for Sustainable Laboratories”. The intention of that policy paper is to provide an evidence-base for investments in the good management of laboratories with a view to sustainable laboratory biosafety and biosecurity.
In addition to this paper, the OIE will enhance the PVS Sustainable Laboratories Tool recognizing that equipment maintenance, repair and calibration are critical components for sustainable laboratory biosafety and biosecurity. The paper will also explore how such investments could encourage local market development to respond to laboratory needs for sustainable laboratory biosafety and biosecurity to mitigate the security and health risks associated with a lack of sustainable resources for veterinary laboratories.

You are requested to collect information on laboratory equipment inventory and maintenance practices from the laboratory where you work. If you don’t work in a laboratory, please send the questionnaire to be filled by the laboratory management of the public veterinary laboratories in your national network. Some questions should be answered about the whole national laboratory network in your country, while others should be answered about the laboratory where you work. The differences between these questions are clearly stated.
Unless otherwise stated, questions should be answered for 2018, or the most recent year for which you have information.
For any issues or for clarification, please contact the Sustainable Laboratories Team for support at b.nyange@oie.int.
Please complete the questionnaire by 17 May 2019.

Definitions
These definitions are proposed for the purposes of this survey.
Maintenance: For the purposes of this survey, 'maintenance' refers to preventive maintenance which is carried out in accordance with a specified time schedule, involving functional checks and servicing, replacement of consumables, etc.
Repair : Corrective maintenance carried out after failure or detection of a fault, in order to restore equipment to working order, including repairing or replacing parts of the equipment
Calibration: Precise adjustments made to laboratory equipment to ensure accurate measurement for a particular function and to establish the metrological traceability of the reported results
Malfunctioning: Equipment not working properly, that may require maintenance, repair, or calibration Local service provider: A service provider within a country
Local service provider: A service provider within a country
External service provider: A service provider outside a country
National laboratory network: All laboratories working in the veterinary domain in a country; for the purposes of this survey, only public laboratories
Central veterinary laboratory: The most advanced laboratory in a country; in some cases, the national reference laboratory of one or more diseases, and often in the administrative capital of a country
On loan equipment: Equipment that is provided by a manufacturer, supplier, university, partner, or other organisation for a limited

Custom Data 1	
**A. Respondent Identification**	
First Name	
Last Name	

Country	
Are you the OIE National Laboratory Focal Point nominated by the OIE Delegate?	
Job Title	
At which level is the veterinary laboratory where you work?	
Other (please specify)	

B. National Laboratory Network

As a Focal Point, Information you provide in this section will allow us to understand the size of your country's national laboratory network.

How many public veterinary laboratories were operating in your national laboratory network in 2018?	
How many public veterinary laboratories are there at the central, federal, or national level in your country?	
ow many public veterinary laboratories are there at the provincial or state level in your country?	
How many public veterinary laboratories are there at the district or local level in your country?	
Are you currently working in a laboratory ?	

Some responses in the questionnaire should be provided by personnel working day-to-day on laboratory resource management and equipment inventory.If you are not currently working in a laboratory, please forward this excelfile to laboratory directors of all public veterinary laboratories in your country so that they may complete the questionnaire. Please feel free to circulate to any and all laboratory management in your national veterinary laboratory network.

At which level is the veterinary laboratory where you work?	
Other (please specify)	
What types of testing/procedures are performed at your laboratory?	

C. Equipment Inventory

Information you provide in this section will allow us to understand the inventory practices in your country's national laboratory network.

How often is the laboratory equipment inventory done per year in your laboratory?	
Do you have "on loan" equipment in your laboratory?	
What percentage of equipment in your laboratory is "on loan"?	
Do you have donated equipment in your inventory?	
What percentage of equipment in your laboratory is donated?	

**D. Barriers to service procurement for laboratory equipment maintenance**	
Information you provide in this section will inform on barriers to procurement of preventive maintenance, calibration, and repair services for laboratory equipment.	
Please choose the **top 3 causes** of malfunctioning or not working equipment that need **repair** in your laboratory	
Over usage of equipment	☐
Software/technology problems of the equipment	☐
Equipment not maintained according to schedule	☐
Power/electricity problems	☐
Improper usage of equipment	☐
Poorly trained personnel using the equipment causing it to break	☐
Physical accidents (dropped, water damage, etc)	☐
Staff don't know how to use, start, or operate	☐
Other	☐
Other (please specify)	
Please choose the **top 3 reasons** why you procure **maintenance** services for laboratory equipment?	
The equipment show signs of malfunction, but is still working	☐
Preventive maintenance is required by law	☐
To keep accreditation/certification status	☐
Routine maintenance is scheduled according to time/date of last service	☐
High demand for analyses linked to particular equipment, so it is important that equipment works properly	☐
To satisfy clients and maintain revenue	☐
To ensure accurate results	☐
Planned/scheduled according to the SOP	☐
Planned/scheduled according to usage/number of analyses run	☐
Other	☐
Other (please specify)	
Please choose the **top 3 reasons** why you procure **calibration** services for laboratory equipment?	
The equipment shows signs of malfunction, but is still working	☐
Calibration is required by law	☐
To keep accreditation/certification status	☐
Routine calibration is scheduled according to time/date of last service	☐

High demand for analyses linked to particular equipment, so it is important that equipment works properly	☐
To satisfy clients and maintain revenue	☐
To ensure accurate results	☐
Planned/scheduled according to the SOP	☐
Planned/scheduled according to usage/number of analyses run	☐
Other	☐
Other (please specify)	

Please choose the **top 3 barriers** you encounter to procuring **maintainance, repair, and calibration** services for your laboratory equipment.	
Services are too expensive	☐
There are no local service providers	☐
Insufficient budget allocated	☐
No in-house expertise	☐
Not prioritized in management activities	☐
Don't know which service providers to use	☐
Expertise is not available	☐
Spare parts are not available	☐
Consummables (filters, etc) are expensive	☐
Procurement of external services is difficult	☐
Other	☐
Other (please specify)	

If you have two pieces of equipment that need to be repaired, but only enough budget to repair one piece of equipment, which **top 3 factors** influence your decision?	
High demand for analyses linked to equipment	☐
Loss of profit if equipment is out of service for long period of time	☐
Loss of capacity to process high sample throughput	☐
Spare parts are available for one and not the other	☐
Age of equipment	☐
Availability of local service provider	☐
Cost of service to repair	☐
Availability of alternative testing method	☐
Other	☐
Other (please specify)	

What do you do with obsolete, damaged, or outdated equipment? (Up to 3 choices possible)	

Put into storage/move out of the laboratory	☐
Label 'Out of Service – Do Not Use'	☐
Isolate equipment in the laboratory	☐
Nothing; staff know it doesn't work	☐
Put in trash/waste	☐
Incinerate	☐
Other	☐
Other (please specify)	

**E. Service Providers**	
Information you provide in this section will help to inform on the service providers you use.	
Do you have a preventive maintenance programme in your laboratory?	
Do you have in-house competencies for equipment maintenance and minor repairs?	
Do you have in-house competencies for calibration of laboratory equipment?	
In your opinion, is there sufficient local service expertise and providers for laboratory equipment maintenance, calibration, and repair in your country?	
In your opinion, what proportion of all maintenance, repair, and calibration services is provided by each of the types of services providers listed below?	
In-house competencies (fill in %)	
Local service providers (fill in %)	
External service providers (fill in %)	
Other (fill in %)	
In your opinion, what percentage of all laboratory equipment in your laboratory is not in proper working order for any reason?	
What is your level of satisfaction with **local** service providers?	
What factors contribute to your satisfaction? (3 choices possible)	
Price	☐
Quality of service	☐
Superior technical expertise	☐
Easy to contact	☐
Available to do work	☐
Donor recommendation/requirement	☐
Proximity	☐
Quick service	☐
Other	☐
Other (please specify)	

What is your level of satisfaction with **external** service providers?	
What factors contribute to your satisfaction? (3 choices possible)	
Price	☐
Quality of service	☐
Superior technical expertise	☐
Easy to contact	☐
Available to do work	☐
Donor recommendation/requirement	☐
Proximity	☐
Quick service	☐
Other	☐
Other (please specify)	

**F. BUDGETING**	
Information you provide in this section will help to understand the budgetary context in which you work and procure services related to laboratory equipment.	
Are maintenance, repair, and calibration services generally included in your annual operational budget?	
What percentage of your annual budget allocation in 2018 was dedicated to equipment maintenance, calibration, and repair? (%)	
In your opinion, is your annual budget allocation for equipment maintenance, calibration, and repair sufficient?	
Who typically funds your laboratory and/or its activities? Check all that apply.	
Government/Veterinary Services	☐
Government/Other	☐
Bilateral donors	☐
Private investors	☐
Non-governmental Organizations (NGO)	☐
International organizations	☐
Other	☐
Other (please specify)	

Who typically funds maintenance, calibration, and repair of laboratory equipment? Check all that apply.	
Government/Veterinary Services	☐
Government/Other	☐

Bilateral donors	☐
Private investors	☐
Non-governmental Organizations (NGO)	☐
International organizations	☐
Other	☐
Other (please specify)	

Thank you for your time spent providing answers to Part 1 of this questionnaire.<br>Please proceed to Part 2 of the questionnaire	

Laboratory Equipment Status Questionnaire for Maintenance/Repair/Calibration - Part I

Background

The main objective of the survey is to assess the status of laboratory equipment maintenance, repair, and calibration for the purposes of a policy paper on "Investment Needs for Sustainable Laboratories". The intention of that policy paper is to provide an evidence-base for investments in the good management of laboratories with a view to sustainable laboratory biosafety and biosecurity.
In addition to this paper, the OIE will enhance the PVS Sustainable Laboratories Tool recognizing that equipment maintenance, repair and calibration are critical components for sustainable laboratory biosafety and biosecurity. The paper will also explore how such investments could encourage local market development to respond to laboratory needs for sustainable laboratory biosafety and biosecurity to mitigate the security and health risks associated with a lack of sustainable resources for veterinary laboratories.

Instructions for the Survey

You are requested to collect information on laboratory equipment inventory and maintenance practices from the laboratory where you work.
If you don't work in a laboratory, please send the questionnaire to be filled by the laboratory management of the public veterinary laboratories in your national network.
Some questions should be answered about the whole national laboratory network in your country, while others should be answered about the laboratory where you work. The differences between these questions are clearly stated.
Unless otherwise stated, questions should be answered for 2018, or the most recent year for which you have information.
For any issues or for clarification, please contact the Sustainable Laboratories Team for support at b.nyange@oie.int.
Please complete the questionnaire by 17 May 2019.

Definitions

These definitions are proposed for the purposes of this survey.
Maintenance: For the purposes of this survey, 'maintenance' refers to preventive maintenance which is carried out in accordance with a specified time schedule, involving functional checks and servicing, replacement of consumables, etc.
Repair : Corrective maintenance carried out after failure or detection of a fault, in order to restore equipment to working order, including repairing or replacing parts of the equipment
Calibration: Precise adjustments made to laboratory equipment to ensure accurate measurement for a particular function and to establish the metrological traceability of the reported results
Malfunctioning: Equipment not working properly, that may require maintenance, repair, or calibration Local service provider: A service provider within a country
Local service provider: A service provider within a country
External service provider: A service provider outside a country
National laboratory network: All laboratories working in the veterinary domain in a country; for the purposes of this survey, only public laboratories
Central veterinary laboratory: The most advanced laboratory in a country; in some cases, the national reference laboratory of one or more diseases, and often in the administrative capital of a country
On loan equipment: Equipment that is provided by a manufacturer, supplier, university, partner, or other organisation for a limited amount of time for trial, testing, or research, while always belonging to the lender.
Donated equipment: Equipment that is given by a partner for an unlimited amount of time and belongs to the beneficiary laboratory.

**A. Respondent Identification**	
First Name	
Last Name	
Country	
Are you the OIE National Laboratory Focal Point nominated by the OIE Delegate?	
Job Title	
At which level is the veterinary laboratory where you work?	
Other (please specify)	

**B. National Laboratory Network**	
As a Focal Point, Information you provide in this section will allow us to understand the size of your country's national laboratory network.	
How many public veterinary laboratories were operating in your national laboratory network in 2018?	
How many public veterinary laboratories are there at the central, federal, or national level in your country?	
How many public veterinary laboratories are there at the provincial or state level in your country?	
How many public veterinary laboratories are there at the district or local level in your country?	
Are you currently working in a laboratory ?	

Some responses in the questionnaire should be provided by personnel working day-to-day on laboratory resource management and equipment inventory.If you are not currently working in a laboratory, please forward this excelfile to laboratory directors of all public veterinary laboratories in your country so that they may complete the questionnaire. Please feel free to circulate to any and all laboratory management in your national veterinary laboratory network.

At which level is the veterinary laboratory where you work?	
Other (please specify)	
What types of testing/procedures are performed at your laboratory?	

C. Equipment Inventory	
Information you provide in this section will allow us to understand the inventory practices in your country's national laboratory network.	
How often is the laboratory equipment inventory done per year in your laboratory?	
Do you have "on loan" equipment in your laboratory?	
What percentage of equipment in your laboratory is "on loan"?	
Do you have donated equipment in your inventory?	
What percentage of equipment in your laboratory is donated?	

D. Barriers to service procurement for laboratory equipment maintenance	
Information you provide in this section will inform on barriers to procurement of preventive maintenance, calibration, and repair services for laboratory equipment.	
Please choose the **top 3 causes** of malfunctioning or not working equipment that need **repair** in your laboratory	
Over usage of equipment	☐
Software/technology problems of the equipment	☐
Equipment not maintained according to schedule	☐
Power/electricity problems	☐
Improper usage of equipment	☐
Poorly trained personnel using the equipment causing it to break	☐
Physical accidents (dropped, water damage, etc)	☐
Staff don't know how to use, start, or operate	☐
Other	☐
Other (please specify)	

Please choose the **top 3 reasons** why you procure **maintenance** services for laboratory equipment?	
The equipment show signs of malfunction, but is still working	☐
Preventive maintenance is required by law	☐
To keep accreditation/certification status	☐
Routine maintenance is scheduled according to time/date of last service	☐
High demand for analyses linked to particular equipment, so it is important that equipment works properly	☐
To satisfy clients and maintain revenue	☐
To ensure accurate results	☐
Planned/scheduled according to the SOP	☐
Planned/scheduled according to usage/number of analyses run	☐
Other	☐
Other (please specify)	

Please choose the **top 3 reasons** why you procure **calibration** services for laboratory equipment?	
The equipment shows signs of malfunction, but is still working	☐
Calibration is required by law	☐
To keep accreditation/certification status	☐
Routine calibration is scheduled according to time/date of last service	☐
High demand for analyses linked to particular equipment, so it is important that equipment works properly	☐
To satisfy clients and maintain revenue	☐
To ensure accurate results	☐
Planned/scheduled according to the SOP	☐
Planned/scheduled according to usage/number of analyses run	☐
Other	☐
Other (please specify)	

Please choose the **top 3 barriers** you encounter to procuring **maintainance, repair, and calibration** services for your laboratory equipment.	
Services are too expensive	☐
There are no local service providers	☐
Insufficient budget allocated	☐
No in-house expertise	☐
Not prioritized in management activities	☐
Don't know which service providers to use	☐
Expertise is not available	☐
Spare parts are not available	☐
Consummables (filters, etc) are expensive	☐
Procurement of external services is difficult	☐
Other	☐
Other (please specify)	

If you have two pieces of equipment that need to be repaired, but only enough budget to repair one piece of equipment, which **top 3 factors** influence your decision?	
High demand for analyses linked to equipment	☐
Loss of profit if equipment is out of service for long period of time	☐
Loss of capacity to process high sample throughput	☐
Spare parts are available for one and not the other	☐
Age of equipment	☐
Availability of local service provider	☐
Cost of service to repair	☐
Availability of alternative testing method	☐
Other	☐
Other (please specify)	

What do you do with obsolete, damaged, or outdated equipment? (Up to 3 choices possible)	
Put into storage/move out of the laboratory	☐
Label 'Out of Service – Do Not Use'	☐
Isolate equipment in the laboratory	☐
Nothing; staff know it doesn't work	☐
Put in trash/waste	☐
Incinerate	☐
Other	☐
Other (please specify)	

**E. Service Providers**	
Information you provide in this section will help to inform on the service providers you use.	
Do you have a preventive maintenance programme in your laboratory?	
Do you have in-house competencies for equipment maintenance and minor repairs?	
Do you have in-house competencies for calibration of laboratory equipment?	
In your opinion, is there sufficient local service expertise and providers for laboratory equipment maintenance, calibration, and repair in your country?	

In your opinion, what proportion of all maintenance, repair, and calibration services is provided by each of the types of services providers listed below?	
In-house competencies (fill in %)	
Local service providers (fill in %)	
External service providers (fill in %)	
Other (fill in %)	
In your opinion, what percentage of all laboratory equipment in your laboratory is not in proper working order for any reason?	

What is your level of satisfaction with **local** service providers?	
What factors contribute to your satisfaction? (3 choices possible)	
Price	☐
Quality of service	☐
Superior technical expertise	☐
Easy to contact	☐
Available to do work	☐
Donor recommendation/requirement	☐
Proximity	☐
Quick service	☐
Other	☐
Other (please specify)	

What is your level of satisfaction with **external** service providers?	
What factors contribute to your satisfaction? (3 choices possible)	
Price	☐
Quality of service	☐
Superior technical expertise	☐
Easy to contact	☐
Available to do work	☐
Donor recommendation/requirement	☐
Proximity	☐
Quick service	☐
Other	☐
Other (please specify)	

**F. BUDGETING**	
Information you provide in this section will help to understand the budgetary context in which you work and procure services related to laboratory equipment.	

Are maintenance, repair, and calibration services generally included in your annual operational budget?	
What percentage of your annual budget allocation in 2018 was dedicated to equipment maintenance, calibration, and repair? (%)	
In your opinion, is your annual budget allocation for equipment maintenance, calibration, and repair sufficient?	

Who typically funds your laboratory and/or its activities? Check all that apply.	
Government/Veterinary Services	☐
Government/Other	☐
Bilateral donors	☐
Private investors	☐
Non-governmental Organizations (NGO)	☐
International organizations	☐
Other	☐
Other (please specify)	

Who typically funds maintenance, calibration, and repair of laboratory equipment? Check all that apply.	
Government/Veterinary Services	☐
Government/Other	☐
Bilateral donors	☐
Private investors	☐
Non-governmental Organizations (NGO)	☐
International organizations	☐
Other	☐
Other (please specify)	

Thank you for your time spent providing answers to Part 1 of this questionnaire.
Please proceed to Part 2 of the questionnaire

Laboratory Equipment Status Questionnaire for Maintenance/Repair/Calibration - Part II

Background

The main objective of the survey is to assess the status of laboratory equipment maintenance, repair, and calibration for the purposes of a policy paper on "Investment Needs for Sustainable Laboratories". The intention of that policy paper is to provide an evidence-base for investments in the good management of laboratories with a view to sustainable laboratory biosafety and biosecurity.
In addition to this paper, the OIE will enhance the PVS Sustainable Laboratories Tool recognizing that equipment maintenance, repair and calibration are critical components for sustainable laboratory biosafety and biosecurity. The paper will also explore how such investments could encourage local market development to respond to laboratory needs for sustainable laboratory biosafety and biosecurity to mitigate the security and health risks associated with a lack of sustainable resources for veterinary laboratories.
The aim for this questionnaire is to collect information on the management of laboratories and laboratory equipment, related to their maintenance, repair, and calibration.

Instructions for the Survey
Laboratory equipment inventory and status information will be required to fill in this questionnaire. Please prepare that information in advance in order to complete the questionnaire.
Questions which cannot be answered during the seminar can be provided in the 7 days following the seminar.
Unless otherwise stated, questions should be answered for 2018 or the most recent year.
For any issues or for clarification, please contact the Sustainable Laboratories Team for support at b.nyange@oie.int. It will take you 15 minutes to complete this survey.
Please fill out this survey by 17 May 2019.

Definitions
These definitions are proposed for the purposes of this survey and are adapted from ISO definitions, where available.
Maintenance: For the purposes of this survey, 'maintenance' refers to preventive maintenance which is carried out in accordance with a specified time schedule, involving functional checks and servicing, replacement of consumables, etc.
Repair : Corrective maintenance carried out after failure or detection of a fault, in order to restore equipment to working order, including repairing or replacing parts of the equipment
Calibration: Precise adjustments made to laboratory equipment to ensure accurate measurement for a particular function and to establish the metrological traceability of the reported results
Malfunctioning: Equipment not working properly, that may require maintenance, repair, or calibration
Local service provider: A service provider within a country
External service provider: A service provider outside a country
National laboratory network: All laboratories working in the veterinary domain in a country; for the purposes of this survey, only public laboratories
Central veterinary laboratory: The most advanced laboratory in a country; in some cases, the national reference laboratory for one or more diseases, and often in the administrative capital of a country

**A. Respondent Identification**	
First Name	
Last Name	
Country	
Are you the OIE National Laboratory Focal Point nominated by the OIE Delegate?	
Job Title	
Are you currently working in a laboratory ?	
Some responses in the questionnaire should be provided by personnel working day-to-day on laboratory resource management and equipment inventory.If you are not currently working in a laboratory, please forward this excelfile to laboratory directors of all public veterinary laboratories in your country so that they may complete the questionnaire. Please feel free to circulate to any and all laboratory management in your national veterinary laboratory network.	
At which level is the veterinary laboratory where you work?	
Other (please specify)	

**B. National Laboratory Network**	
Information you provide in this section will allow us to understand the size of your country's national laboratory network.	
How many laboratories in your national laboratory network have obtained accreditation to ISO 17025 for at least one test?	
How many laboratories in your national laboratory network have a dedicated Quality Manager?	
In your opinion, what percentage of all laboratory equipment in your national veterinary laboratory network is not in proper working order for any reason?	
In your opinion, what percentage of the annual budget should be allocated for the maintenance, calibration, and repair of equipment?	

**C. BUDGETING**	
In your opinion, what are the **top 3 reasons** why you lack budget for equipment maintenance, repair, and calibration services?	
Administration doesn't understand importance of equipment maintenance, calibration, and repair to Human and Animal Health	☐
Insufficient budget for operating costs of the laboratory in general, let alone for equipment maintenance	☐
Too many competing budget priorities	☐
No "culture" for maintenance, repair and calibration in my laboratory or country	☐
Administration doesn't understand importance of equipment maintenance, calibration, and repair to biosafety and biosecurity	☐
Administration doesn't understand importance of equipment maintenance, calibration, and repair to trade	☐
Expectation/Experience that others will provide resources for equipment maintenance, calibration, and repair	☐

Other (please specify)	

In your organisation's procedures and rules, do you have requirements for service provision on the local market for laboratory equipment maintenance, calibration, or repair ?	
**D. General Laboratory Equipment Inventory**	

Instructions for the next section
The next section of the questionnaire addresses your laboratory equipment status. We will ask questions about each type of general equipment in your laboratory. Please fill in the relevant number of each equipment.

Do you have Biological **Safety Cabinets – Class I** in your laboratory?	
Please provide the relevant number of ***biosafety cabinets – class I*** *in your laboratory.*	
How many do you have in your laboratory?	
How many are in good working order ?	
How many are not working and need minor repair ?	
How many are obsolete or the technology is outdated?	
How many not working and need major repair ?	
How many are properly certified by an accredited service provider?	
How many have been properly calibrated?	
Is there a local service provider(s) available who can maintain, calibrate, or repair this equipment type?	
Is there a local service provider(s) available who can maintain, calibrate, or repair this equipment type?	
Do you have an in-house competency available who can maintain, calibrate, or repair this equipment type?	

Do you have Biological Safety **Cabinet - Class II A1** in your laboratory ?	
Please provide the relevant number of **Biological Safety Cabinet - class II A1** in your laboratory.	
How many do you have in your laboratory?	
How many are in good working order ?	
How many are not working and need minor repair ?	
How many are obsolete or the technology is outdated?	
How many not working and need major repair ?	
How many are properly certified by an accredited service provider?	
How many have been properly calibrated?	
Is there a local service provider(s) available who can maintain, calibrate, or repair this equipment type?	
Is there a local service provider(s) available who can maintain, calibrate, or repair this equipment type?	
Do you have an in-house competency available who can maintain, calibrate, or repair this equipment type?	

Do you have any **Biological Safety Cabinet - class II A2** in your laboratory ?	
Please provide the relevant number of ***Biological Safety Cabinet - class II A2*** *in your laboratory.*	
How many do you have in your laboratory?	
How many are in good working order ?	
How many are not working and need minor repair ?	
How many are obsolete or the technology is outdated?	
How many not working and need major repair ?	
How many are properly certified by an accredited service provider?	
How many have been properly calibrated?	
Is there a local service provider(s) available who can maintain, calibrate, or repair this equipment type?	
Is there a local service provider(s) available who can maintain, calibrate, or repair this equipment type?	
Do you have an in-house competency available who can maintain, calibrate, or repair this equipment type?	

Do you have any **Fume hoods** in your laboratory ?	
Please provide the relevant number of ***Fume hoods*** *in your laboratory.*	
How many do you have in your laboratory?	
How many are in good working order ?	
How many are not working and need minor repair ?	
How many are obsolete or the technology is outdated?	
How many not working and need major repair ?	
How many have been properly maintained ?	
How many have been properly calibrated?	
Is there a local service provider(s) available who can maintain, calibrate, or repair this equipment type?	
Do you have an in-house competency available who can maintain, calibrate, or repair this equipment type?	

Do you have any type of **Incubator** (basic, cooled, humidity, shaking, hybridisation) in your laboratory ?	
Please provide the relevant number of **Incubators** (basic, cooled, humidity, shaking, hybridisation) in your laboratory.	
How many do you have in your laboratory?	
How many are in good working order ?	
How many are not working and need minor repair ?	
How many are obsolete or the technology is outdated?	
How many not working and need major repair ?	
How many have been properly maintained ?	
How many have been properly calibrated?	
Is there a local service provider(s) available who can maintain, calibrate, or repair this equipment type?	
Do you have an in-house competency available who can maintain, calibrate, or repair this equipment type?	
Do you have any type of **Autoclave** (basic, waste) in your laboratory ?	
Please provide the relevant number of **Autoclaves** (basic, waste) in your laboratory.	
How many do you have in your laboratory?	
How many are in good working order ?	
How many are not working and need minor repair ?	
How many are obsolete or the technology is outdated?	
How many not working and need major repair ?	
How many have been properly maintained ?	
How many have been properly calibrated?	
Is there a local service provider(s) available who can maintain, calibrate, or repair this equipment type?	
Do you have an in-house competency available who can maintain, calibrate, or repair this equipment type?	
Do you have any type of **Pipette** (automatic, monochannel or multichannel) in your laboratory ?	
*Please provide the relevant number of **Pipettes** (automatic, monochannel or multichannel) in your laboratory.*	
How many do you have in your laboratory?	
How many are in good working order ?	
How many are not working and need minor repair ?	
How many are obsolete or the technology is outdated?	
How many not working and need major repair ?	
How many have been properly maintained ?	
How many have been properly calibrated?	
Is there a local service provider(s) available who can maintain, calibrate, or repair this equipment type?	
Do you have an in-house competency available who can maintain, calibrate, or repair this equipment type?	
Do you have any type of **Centrifuge or microcentrifuge** (plates, tubes, refrigerated) in your laboratory ?	
*Please provide the relevant number of **Centrifuges or microcentrifuges** (plates, tubes, refrigerated) in your laboratory.*	
How many do you have in your laboratory?	
How many are in good working order ?	
How many are not working and need minor repair ?	
How many are obsolete or the technology is outdated?	
How many not working and need major repair ?	
How many have been properly maintained ?	
How many have been properly calibrated?	
Is there a local service provider(s) available who can maintain, calibrate, or repair this equipment type?	
Do you have an in-house competency available who can maintain, calibrate, or repair this equipment type?	
Do you have **Refrigerators** in your laboratory ?	
*Please provide the relevant number of **Refrigerators** in your laboratory.*	
How many do you have in your laboratory?	
How many are in good working order ?	
How many are not working and need minor repair ?	
How many are obsolete or the technology is outdated?	
How many not working and need major repair ?	
How many have been properly maintained ?	
How many have been properly calibrated?	
Is there a local service provider(s) available who can maintain, calibrate, or repair this equipment type?	
Do you have an in-house competency available who can maintain, calibrate, or repair this equipment type?	

Do you have a **Water filtration system** in your laboratory ?	
Please provide the relevant number of ***Water filtration systems*** *in your laboratory.*	
How many do you have in your laboratory?	
How many are in good working order ?	
How many are not working and need minor repair ?	
How many are obsolete or the technology is outdated?	
How many not working and need major repair ?	
How many have been properly maintained ?	
How many have been properly calibrated?	
Is there a local service provider(s) available who can maintain, calibrate, or repair this equipment type?	
Do you have an in-house competency available who can maintain, calibrate, or repair this equipment type?	

Do you have any type of **Freezer** (-20°C and -80°) in your laboratory ?	
Please provide the relevant number of ***Freezers*** *(-20°C and -80°) in your laboratory.*	
How many do you have in your laboratory?	
How many are in good working order ?	
How many are not working and need minor repair ?	
How many are obsolete or the technology is outdated?	
How many not working and need major repair ?	
How many have been properly maintained ?	
How many have been properly calibrated?	
Is there a local service provider(s) available who can maintain, calibrate, or repair this equipment type?	
Do you have an in-house competency available who can maintain, calibrate, or repair this equipment type?	

Do you have any **Ovens** in your laboratory ?	
Please provide the relevant number of ***Ovens*** *in your laboratory.*	
How many do you have in your laboratory?	
How many are in good working order ?	
How many are not working and need minor repair ?	
How many are obsolete or the technology is outdated?	
How many not working and need major repair ?	
How many have been properly maintained ?	
How many have been properly calibrated?	
Is there a local service provider(s) available who can maintain, calibrate, or repair this equipment type?	
Is there a local service provider(s) available who can maintain, calibrate, or repair this equipment type?	
Do you have an in-house competency available who can maintain, calibrate, or repair this equipment type?	

Do you have a **Colony counter** in your laboratory ?	
Please provide the relevant number of ***Colony counters*** *in your laboratory.*	
How many do you have in your laboratory?	
How many are in good working order ?	
How many are not working and need minor repair ?	
How many are obsolete or the technology is outdated?	
How many not working and need major repair ?	
How many have been properly maintained ?	
How many have been properly calibrated?	
Is there a local service provider(s) available who can maintain, calibrate, or repair this equipment type?	
Do you have an in-house competency available who can maintain, calibrate, or repair this equipment type?	

Do you have a **Gas incubator** in your laboratory ?	
Please provide the relevant number of ***Gas incubators*** *in your laboratory.*	
How many do you have in your laboratory?	
How many are in good working order ?	
How many are not working and need minor repair ?	
How many are obsolete or the technology is outdated?	
How many not working and need major repair ?	
How many have been properly maintained ?	
How many have been properly calibrated?	
Is there a local service provider(s) available who can maintain, calibrate, or repair this equipment type?	
Do you have an in-house competency available who can maintain, calibrate, or repair this equipment type?	

Do you have a **Dark field microscope** in your laboratory ?	
Please provide the relevant number of ***Dark field microscopes*** *in your laboratory.*	
How many do you have in your laboratory?	
How many are in good working order ?	

How many are not working and need minor repair ?	
How many are obsolete or the technology is outdated?	
How many not working and need major repair ?	
How many have been properly maintained ?	
How many have been properly calibrated?	
Is there a local service provider(s) available who can maintain, calibrate, or repair this equipment type?	
Is there a local service provider(s) available who can maintain, calibrate, or repair this equipment type?	
Do you have an in-house competency available who can maintain, calibrate, or repair this equipment type?	

Do you have a **Microtome** in your laboratory ?	
*Please provide the relevant number of **Microtomes** in your laboratory.*	
How many do you have in your laboratory?	
How many are in good working order ?	
How many are not working and need minor repair ?	
How many are obsolete or the technology is outdated?	
How many not working and need major repair ?	
How many have been properly maintained ?	
How many have been properly calibrated?	
Is there a local service provider(s) available who can maintain, calibrate, or repair this equipment type?	
Do you have an in-house competency available who can maintain, calibrate, or repair this equipment type?	

Do you have a **Fluorescent microscop**e in your laboratory ?	
*Please provide the relevant number of **Fluorescent microscope** s in your laboratory.*	
How many do you have in your laboratory?	
How many are in good working order ?	
How many are not working and need minor repair ?	
How many are obsolete or the technology is outdated?	
How many not working and need major repair ?	
How many have been properly maintained ?	
How many have been properly calibrated?	
Is there a local service provider(s) available who can maintain, calibrate, or repair this equipment type?	
Do you have an in-house competency available who can maintain, calibrate, or repair this equipment type?	

Do you have a **Mixer jar** in your laboratory ?	
*Please provide the relevant number of **Mixer jars** in your laboratory.*	
How many do you have in your laboratory?	
How many are in good working order ?	
How many are not working and need minor repair ?	
How many are obsolete or the technology is outdated?	
How many not working and need major repair ?	
How many have been properly maintained ?	
How many have been properly calibrated?	
Is there a local service provider(s) available who can maintain, calibrate, or repair this equipment type?	
Do you have an in-house competency available who can maintain, calibrate, or repair this equipment type?	

Do you have a **Trichinoscope** in your laboratory ?	
Please provide the relevant number of ***Trichinoscopes*** in your laboratory.	
How many do you have in your laboratory?	
How many are in good working order ?	
How many are not working and need minor repair ?	
How many are obsolete or the technology is outdated?	
How many not working and need major repair ?	
How many have been properly maintained ?	
How many have been properly calibrated?	
Is there a local service provider(s) available who can maintain, calibrate, or repair this equipment type?	
Is there a local service provider(s) available who can maintain, calibrate, or repair this equipment type?	
Do you have an in-house competency available who can maintain, calibrate, or repair this equipment type?	

Do you have a Transilluminator in your laboratory ?	
Please provide the relevant number of Transilluminators in your laboratory.	
How many do you have in your laboratory?	
How many are in good working order ?	
How many are not working and need minor repair ?	
How many are obsolete or the technology is outdated?	

How many not working and need major repair ?	
How many have been properly maintained ?	
How many have been properly calibrated?	
Is there a local service provider(s) available who can maintain, calibrate, or repair this equipment type?	
Do you have an in-house competency available who can maintain, calibrate, or repair this equipment type?	
Do you have **Real-time PCR** in your laboratory ?	
Please provide the relevant number of ***Real-time PCRs*** *in your laboratory.*	
How many do you have in your laboratory?	
How many are in good working order ?	
How many are not working and need minor repair ?	
How many are obsolete or the technology is outdated?	
How many not working and need major repair ?	
How many have been properly maintained ?	
How many have been properly calibrated?	
Is there a local service provider(s) available who can maintain, calibrate, or repair this equipment type?	
Do you have an in-house competency available who can maintain, calibrate, or repair this equipment type?	
Do you have a **Thermal cycler/conventional PCR** in your laboratory ?	
Please provide the relevant number of ***Thermal cycler/conventional PCRs*** *in your laboratory.*	
How many do you have in your laboratory?	
How many are in good working order ?	
How many are not working and need minor repair ?	
How many are obsolete or the technology is outdated?	
How many not working and need major repair ?	
How many have been properly maintained ?	
How many have been properly calibrated?	
Is there a local service provider(s) available who can maintain, calibrate, or repair this equipment type?	
Do you have an in-house competency available who can maintain, calibrate, or repair this equipment type?	
Do you have a **Electrophoresis machine/power supply** in your laboratory ?	
Please provide the relevant number of ***Electrophoresis machine/power supply*** *in your laboratory.*	
How many do you have in your laboratory?	
How many are in good working order ?	
How many are not working and need minor repair ?	
How many are obsolete or the technology is outdated?	
How many not working and need major repair ?	
How many have been properly maintained ?	
How many have been properly calibrated?	
Is there a local service provider(s) available who can maintain, calibrate, or repair this equipment type?	
Do you have an in-house competency available who can maintain, calibrate, or repair this equipment type?	
Do you have a **Gel documentation** system in your laboratory ?	
Please provide the relevant number of ***Gel documentation systems*** *in your laboratory.*	
How many do you have in your laboratory?	
How many are in good working order ?	
How many are not working and need minor repair ?	
How many are obsolete or the technology is outdated?	
How many not working and need major repair ?	
How many have been properly maintained ?	
How many have been properly calibrated?	
Is there a local service provider(s) available who can maintain, calibrate, or repair this equipment type?	
Is there a local service provider(s) available who can maintain, calibrate, or repair this equipment type?	
Do you have an in-house competency available who can maintain, calibrate, or repair this equipment type?	
Do you have a **Microplate reader** and dedicated computer in your laboratory ?	
Please provide the relevant number of ***Microplate readers*** *& dedicated computer in your laboratory.*	
How many do you have in your laboratory?	
How many are in good working order ?	
How many are not working and need minor repair ?	
How many are obsolete or the technology is outdated?	
How many not working and need major repair ?	

How many have been properly maintained ?	
How many have been properly calibrated?	
Is there a local service provider(s) available who can maintain, calibrate, or repair this equipment type?	
Do you have an in-house competency available who can maintain, calibrate, or repair this equipment type?	
Do you have a **Microplate washe**r in your laboratory ?	
*Please provide the relevant number of **Microplate washers** in your laboratory.*	
How many do you have in your laboratory?	
How many are in good working order ?	
How many are not working and need minor repair ?	
How many are obsolete or the technology is outdated?	
How many not working and need major repair ?	
How many have been properly maintained ?	
How many have been properly calibrated?	
Is there a local service provider(s) available who can maintain, calibrate, or repair this equipment type?	
Is there a local service provider(s) available who can maintain, calibrate, or repair this equipment type?	
Do you have an in-house competency available who can maintain, calibrate, or repair this equipment type?	
Do you have a **Plate shaker** in your laboratory ?	
*Please provide the relevant number of **Plate shakers** in your laboratory.*	
How many do you have in your laboratory?	
How many are in good working order ?	
How many are not working and need minor repair ?	
How many are obsolete or the technology is outdated?	
How many not working and need major repair ?	
How many have been properly maintained ?	
How many have been properly calibrated?	
Is there a local service provider(s) available who can maintain, calibrate, or repair this equipment type?	
Do you have an in-house competency available who can maintain, calibrate, or repair this equipment type?	
Do you have an **Inverted light microscope** in your laboratory ?	
*Please provide the relevant number of **Inverted light microscopes** in your laboratory.*	
How many do you have in your laboratory?	
How many are in good working order ?	
How many are not working and need minor repair ?	
How many are obsolete or the technology is outdated?	
How many not working and need major repair ?	
How many have been properly maintained ?	
How many have been properly calibrated?	
Is there a local service provider(s) available who can maintain, calibrate, or repair this equipment type?	
Do you have an in-house competency available who can maintain, calibrate, or repair this equipment type?	
Do you have a **Roller system** in your laboratory ?	
*Please provide the relevant number of **Roller systems** in your laboratory.*	
How many do you have in your laboratory?	
How many are in good working order ?	
How many are not working and need minor repair ?	
How many are obsolete or the technology is outdated?	
How many not working and need major repair ?	
How many have been properly maintained ?	
How many have been properly calibrated?	
Is there a local service provider(s) available who can maintain, calibrate, or repair this equipment type?	
Is there a local service provider(s) available who can maintain, calibrate, or repair this equipment type?	
Do you have an in-house competency available who can maintain, calibrate, or repair this equipment type?	
Do you have a **Conductometer** in your laboratory ?	
Please provide the relevant number of ***Conductometers*** in your laboratory.	
How many do you have in your laboratory?	
How many are in good working order ?	
How many are not working and need minor repair ?	
How many are obsolete or the technology is outdated?	
How many not working and need major repair ?	
How many have been properly maintained ?	
How many have been properly calibrated?	

Is there a local service provider(s) available who can maintain, calibrate, or repair this equipment type?	
Do you have an in-house competency available who can maintain, calibrate, or repair this equipment type?	
Do you have a **Distillator** in your laboratory ?	
Please provide the relevant number of ***Distillators*** *in your laboratory.*	
How many do you have in your laboratory?	
How many are in good working order ?	
How many are not working and need minor repair ?	
How many are obsolete or the technology is outdated?	
How many not working and need major repair ?	
How many have been properly maintained ?	
How many have been properly calibrated?	
Is there a local service provider(s) available who can maintain, calibrate, or repair this equipment type?	
Do you have an in-house competency available who can maintain, calibrate, or repair this equipment type?	
Do you have a **Magnetic agitator** in your laboratory ?	
Please provide the relevant number of ***Magnetic agitators*** *in your laboratory.*	
How many do you have in your laboratory?	
How many are in good working order ?	
How many are not working and need minor repair ?	
How many are obsolete or the technology is outdated?	
How many not working and need major repair ?	
How many have been properly maintained ?	
How many have been properly calibrated?	
Is there a local service provider(s) available who can maintain, calibrate, or repair this equipment type?	
Is there a local service provider(s) available who can maintain, calibrate, or repair this equipment type?	
Do you have an in-house competency available who can maintain, calibrate, or repair this equipment type?	
Do you have a **Microscope and camera** in your laboratory ?	
Please provide the relevant number of ***Microscopes and camera*** *in your laboratory.*	
How many do you have in your laboratory?	
How many are in good working order ?	
How many are not working and need minor repair ?	
How many are obsolete or the technology is outdated?	
How many not working and need major repair ?	
How many have been properly maintained ?	
How many have been properly calibrated?	
Is there a local service provider(s) available who can maintain, calibrate, or repair this equipment type?	
Do you have an in-house competency available who can maintain, calibrate, or repair this equipment type?	
Do you have an **Ovoscope** in your laboratory ?	
Please provide the relevant number of ***Ovoscopes*** *in your laboratory.*	
How many do you have in your laboratory?	
How many are in good working order ?	
How many are not working and need minor repair ?	
How many are obsolete or the technology is outdated?	
How many not working and need major repair ?	
How many have been properly maintained ?	
How many have been properly calibrated?	
Is there a local service provider(s) available who can maintain, calibrate, or repair this equipment type?	
Do you have an in-house competency available who can maintain, calibrate, or repair this equipment type?	
Do you have a **pH meter** in your laboratory ?	
Please provide the relevant number of ***pH meters*** *in your laboratory.*	
How many do you have in your laboratory?	
How many are in good working order ?	
How many are not working and need minor repair ?	
How many are obsolete or the technology is outdated?	
How many not working and need major repair ?	
How many have been properly maintained ?	
How many have been properly calibrated?	
Is there a local service provider(s) available who can maintain, calibrate, or repair this equipment type?	
Is there a local service provider(s) available who can maintain, calibrate, or repair this equipment type?	

Do you have an in-house competency available who can maintain, calibrate, or repair this equipment type?	
Do you have any type of **Shaker** (oscillator/rotative/orbital) in your laboratory ?	
Please provide the relevant number of ***Shakers*** *(oscillator/rotative/orbital) in your laboratory.*	
How many do you have in your laboratory?	
How many are in good working order ?	
How many are not working and need minor repair ?	
How many are obsolete or the technology is outdated?	
How many not working and need major repair ?	
How many have been properly maintained ?	
How many have been properly calibrated?	
Is there a local service provider(s) available who can maintain, calibrate, or repair this equipment type?	
Do you have an in-house competency available who can maintain, calibrate, or repair this equipment type?	
Do you have a **Spectrophotomete**r – basic in your laboratory ?	
Please provide the relevant number of ***Spectrophotometer*** *s – basic in your laboratory.*	
How many do you have in your laboratory?	
How many are in good working order ?	
How many are not working and need minor repair ?	
How many are obsolete or the technology is outdated?	
How many not working and need major repair ?	
How many have been properly maintained?	
How many have been properly calibrated?	
Is there a local service provider(s) available who can maintain, calibrate, or repair this equipment type?	
Do you have an in-house competency available who can maintain, calibrate, or repair this equipment type?	
Do you have a **Vacuum pump i**n your laboratory ?	
Please provide the relevant number of ***Vacuum pumps*** *in your laboratory.*	
How many do you have in your laboratory?	
How many are in good working order ?	
How many are not working and need minor repair ?	
How many are obsolete or the technology is outdated?	
How many not working and need major repair ?	
How many have been properly maintained ?	
How many have been properly calibrated?	
Is there a local service provider(s) available who can maintain, calibrate, or repair this equipment type?	
Do you have an in-house competency available who can maintain, calibrate, or repair this equipment type?	
Do you have a **Vortex** in your laboratory ?	
Please provide the relevant number of ***Vortex*** *in your laboratory.*	
How many do you have in your laboratory?	
How many are in good working order ?	
How many are not working and need minor repair ?	
How many are obsolete or the technology is outdated?	
How many not working and need major repair ?	
How many have been properly maintained ?	
How many have been properly calibrated?	
Is there a local service provider(s) available who can maintain, calibrate, or repair this equipment type?	
Do you have an in-house competency available who can maintain, calibrate, or repair this equipment type?	
Do you have a **Water bath** in your laboratory ?	
Please provide the relevant number of **Water baths** in your laboratory.	
How many do you have in your laboratory?	
How many are in good working order ?	
How many are not working and need minor repair ?	
How many are obsolete or the technology is outdated?	
How many not working and need major repair ?	
How many have been properly maintained ?	
How many have been properly calibrated?	
Is there a local service provider(s) available who can maintain, calibrate, or repair this equipment type?	
Is there a local service provider(s) available who can maintain, calibrate, or repair this equipment type?	
Do you have an in-house competency available who can maintain, calibrate, or repair this equipment type?	

**G. Cost of maintenance, repair and calibration services**	
Please collect 3 invoices of three pieces of equipment listed here which were maintained, calibrated, or repaired in the past 10 years.	
**INVOICE 1**	
Please select an equipment	
In what year was the equipment acquired?	
What was the nature of the service provided?	
Date of service	
Cost of service (USD)	
Type of Service Provider that performed the service.	
Country of origin of service provider	
**INVOICE 2**	
Please select an equipment	
In what year was the equipment acquired?	
What was the nature of the service provided?	
Date of service	
Cost of service (USD)	
Type of Service Provider that performed the service.	
Country of origin of service provider	
**INVOICE 3**	
Please select an equipment	
In what year was the equipment acquired?	
What was the nature of the service provided?	
Date of service	
Cost of service (USD)	
Type of Service Provider that performed the service.	
Country of origin of service provider	

Thank you for your time spent providing answers to this questionnaire.
